# Supplementary material for: Knowledge, attitudes, and practices of healthcare professionals regarding rabies in tertiary care hospitals: A cross-sectional study in Peshawar, Pakistan
Source: PLoS Negl Trop Dis. 2024 Jun 10;18(6):e0012238. doi: 10.1371/journal.pntd.0012238 (PMC11192319; doi:10.1371/journal.pntd.0012238)
Supplement: Attachment — Submitted filename: Adressing Comments.docx [file pntd.0012238.s002.docx]

**Subject:** Cover Letter for Revised Research Article Submission

Dear Prof. Dr. Peter J. Hotez,

I hope this letter finds you well. I am writing to submit the revised version of our research article entitled "Knowledge, Attitudes, and Practices of Healthcare Professionals Regarding Rabies in Tertiary Care Hospitals: A Cross-Sectional Study in Peshawar, Pakistan" for your kind consideration.

We sincerely appreciate the time and effort invested by the reviewers in providing constructive feedback, which has significantly contributed to enhancing the quality and clarity of our manuscript. In response to the valuable comments received, we have made meticulous revisions to address each point raised.

The revisions include refinements in the description of rabies endemicity, clarification of statistical correlations, and improvements in the overall structure and scientific language of the manuscript. We believe that these modifications have strengthened the robustness of our study and its contribution to the understanding of rabies awareness among healthcare professionals.

We are confident that the revised manuscript aligns with the high standards of Plos Neglected Tropical Diseases and meets the criteria for publication. We would be honored if you would consider our submission for further review.

Thank you for your time, consideration, and commitment to advancing scientific knowledge in the field. We look forward to the opportunity of having our research published in Plos Neglected Tropical Diseases.

**Sincerely,**

**Dr Shehnaz Bakhtiar**

**bakhtiarshehnaz@gmail.com**

**Knowledge, Attitudes, and Practices of Healthcare Professionals Regarding Rabies in Tertiary Care Hospitals: A Cross-Sectional Study in Peshawar, Pakistan**

Adnan Ahmad^1^, Fawad Inayat^2^, Noor Ullah^3^, Shaista rasul^4^, Shehnaz Bakhtiar^5*^, Zar Shad^6^, Zakir Ahmad^2^

^1^Department of Allied Health Sciences, Cecos University of IT and Emerging Sciences Peshawar Pakistan

^2^Department of Biochemistry, Institute of Chemical and Life Science, Abdul Wali Khan University Mardan, Pakistan.

^3^Institute of Paramedical Sciences, Khyber Medical University, Peshawar, Pakistan.

^4^Institute of Public Health and Social Sciences, Khyber Medical University Peshawar, Pakistan.

^5^Khyber Institute of Child Health and Bashir Bilour Memorial Children Hospital, Peshawar, Pakistan.

^6^Department of Biological and Health Sciences, Abasyn University, Peshawar, Pakistan.

Corresponding Author: Dr Shehnaz Bakhtiar

Email address: [Bakhtiarshehnaz@gmail.com](mailto:Bakhtiarshehnaz@gmail.com),

Phone number: +923130918155

Note: The reviewer one comments were highlighted in yellow in main text, reviewer two comments in light blue and reviewer three comments in light green.

**Reviewer #1: The title and summary are concise and clear.**

Line 36 - It was not clear to which places the authors are referring regarding the incidence rate of rabies-related deaths:

In Peshawar, Pakistan.

India - 20 to 30 cases per million per year.

Not described (Bangladesh?) - 14 cases per million per year.

Yes, it is in Bangladesh.

Pakistan - 7 to 9 cases per million per year

Yes, as it is mentioned in.

Methodology – It was not described which health professionals were included in the sample.

Cross-sectional research was conducted to gather data from 100 healthcare workers representing different sections within the healthcare field, including Medical Officers, House Officers, Faculty Staff, Nurses, and Paramedics.

The described methodology supports the achievement of the proposed objectives.

The research was conducted after approval by the ethics committee and with healthcare professionals' consent in Pakistan's hospitals.

**Reviewer #2: The article “Knowledge, Attitudes, and Practices of Healthcare Professionals Regarding Rabies in Tertiary Care Hospitals: A Cross-Sectional Study in Peshawar, Pakistan” addresses an important topic but I have following concerns,**

1. The introduction doesn’t provide sufficient background in the topic of research and the objectives are not clearly defined.

Made possible correction in the introduction.

2. The study duration is missing only a period of 6 months has been mentioned.

16 August 2021 to 15 February 2022

3. The authors have not mentioned how they have collected the sample size.

The sample size was based on the previous published study and I have mentioned the necessary references.

4. The ethical approval number is missing.

DIR/KMU-EB/MS/00073

5. No details are provided regarding the questionnaire used for the study and what was the language of the questionnaire and how it was validated?

The structured questionnaire was derived from previous KAP surveys done worldwide on rabies i.e., Sri Lanka, Pakistan, and India. (I have mentioned the references).

**Reviewer #3: I have carefully reviewed the Methods section of your research manuscript. Overall, the study design appears to be robust, but there are some discrepancies and areas that require further clarification. Below is a detailed analysis of the comments provided:**

Design Clarification:
The term "design" should be explicitly defined in the Methods section. Please provide a clear and detailed explanation of the study design employed.

The term “designed” was excluded as we have used a standard questionnaire from a Sri Lankan Study.

Separation of Questionnaire and Consent:
It is recommended to remove the questionnaire and consent from the main text in the study design and present them separately. This enhances clarity and allows readers to focus on the research methods without distraction.

Done as directed.

Duration of the Study:
The duration of the study should be precisely stated in terms of start and end dates. Remove unnecessary wording and provide an exact time frame for the study period.

16 August 2021 to 15 February 2022

Hospital Selection and Sample Size Calculation:
Clarify the criteria used for selecting the participating hospitals. Additionally, elaborate on how the sample size was determined, addressing issues related to statistical power and whether the study meets the required power for significance testing.

All the tertiary care Government hospitals that provide rabies treatment in Peshawar were included in the study. Moreover, the sample size was based on the previously published paper.

Languages Used in Questionnaire:
Specify the languages in which the questionnaire was conducted. Additionally, explain the process of translating questions from other studies into the required languages. This information is crucial for understanding the linguistic validity of the instrument.

The Questionnaire used was in English and comprised of four sections. Moreover, all the participants understand the English language.

Sampling Technique and Sample Selection:
Explicitly state the sampling technique employed in the study. Provide details on how participants were selected, ensuring transparency in the sampling process.

Sampling technique and sample selection were on the convenient sampling technique.

Ethical Board Approval and Questionnaire Reference:
Include the ethical board approval number and reference for the questionnaires used in your study. This adds credibility to your research and ensures compliance with ethical standards.

DIR/KMU-EB/MS/00073. Moreover, I have provided the references for the questionnaire.

Survey Language:
Clearly state whether the survey was conducted in English, Urdu, or both. Additionally, provide more details on the items and sections of the questionnaire to give readers a comprehensive understanding of the data collection process.

The Questionnaire used was in English and Comprises of four sections.

1). Demographic 2). Knowledge 3). Attitude 4). Practices.

Data Analysis Description:
Elaborate on the data analysis methods used in detail. Include any specific procedures for handling missing data and statistical test used. This will enhance the transparency and reproducibility of your findings.

Actually, we have excluded those questionnaires that are missing with the aimed parameters or questions and thus avoid any missing while entering data into MS Excel and SPSS.

**Result**

**Reviewer #1:** The acronym (ARV) in table 2 is wrong:
Knowledge regarding availability of anti-rabies vaccine (AVR) in the vicinity of study hospitals.

Corrected as directed.
In table 3, the values related to the variable “Rabies can spread through licks scratches” are wrong.

Corrected accordingly.

**Reviewer #3:** I have carefully reviewed the manuscript, and I would like to provide the following comments for your consideration:

Deletion of First Two Lines (154, 155):
I suggest removing the first two lines (154, 155) as they appear redundant or unnecessary in the context of the manuscript.

Done as directed.

Revision of Sentences (157-159):
The sentences in this section give the impression that the survey is conducted in a single hospital. Please consider revising these sentences to accurately convey the scope and setting of the survey to avoid potential misinterpretations.

Done as directed.

Deletion of a Sentence (Regarding Gender Inequalities):
I recommend removing the sentence that discusses possible ramifications for the findings of the research due to pre-existing gender inequalities in the healthcare sector. This statement appears to be out of context and doesn't contribute to the clarity of the manuscript.

Done as directed.

Overall Results Revision:
The overall results section needs a comprehensive revision for improved clarity. Consider reorganizing and rewriting this section from scratch to enhance the readability and coherence of the presented information. Providing a clear and concise summary of the research findings will significantly strengthen the manuscript.

Done as directed and have completely revised the result section and made changes accordingly.

Ambiguity in the Term "Training" (Table 1):
The term "Training" in Table 1 is deemed ambiguous. I recommend clearly defining or specifying the type of training being referred to in the table to eliminate any potential confusion for readers. Additionally, please ensure the table is formatted more clearly, and consider removing the percentage sign for better presentation.

All the participants were asked about their training as the training was about the handling of rabies infection and rabies-infected individuals according to the WHO guidelines.

Results Section Revamp:
The results section, as it stands, was challenging to follow. A complete revision is necessary to enhance the clarity and coherence of the information presented. Provide a concise and organized summary of the research findings, ensuring that the narrative is logically structured and easy to comprehend.

Revised all the results accordingly.

**Conclusion**

**Reviewer #1:** The conclusions are supported by the data presented, corroborating similar studies in other locations with similar contexts.

**Reviewer #3:** NA

**Editorial and Data Presentation Modifications?**

**Reviewer #1:** Minor Revision

**Reviewer #2:** (No Response)

**Reviewer #3:** Before sending the manuscript for peer review, the editorial desk should conduct a careful check of its worth and scope, language and structure, as well as its conformity to the journal guidelines.

Done as directed.

**Summary and General Comments**
Use this section to provide overall comments, discuss strengths/weaknesses of the study, novelty, significance, general execution and scholarship. You may also include additional comments for the author, including concerns about dual publication, research ethics, or publication ethics. If requesting major revision, please articulate the new experiments that are needed.

Reviewer #1: Rabies is a disease that is essentially 100% lethal, yet 100% preventable through the implementation of appropriate and timely control measures. This study aimed to assess the knowledge, attitudes, and practices of healthcare professionals working in the emergency sector of three tertiary hospitals. The survey results hold significance as they provide insights into the rabies knowledge, attitudes, and practices of emergency professionals in Pakistan, a region with a high rabies mortality rate.
It would be valuable to briefly discuss the variance in training among different professional categories, as the information they possess may vary based on their level of education and expertise.
The authors might consider exploring the significance of each professional's role in providing care to individuals at risk of rabies. Among the professionals included in the sample, who plays the most crucial role in prescribing prophylaxis through vaccination, immunoglobulin administration, wound care, treatment, and the notification of suspected human rabies cases?
We recommend including an overview of these hospitals' impact on the local healthcare network. Are these hospitals primarily responsible for rabies care?
It would be enlightening to understand what proportion of hospitals provide similar care and whether these hospitals serve as reference points for individuals exposed to the rabies virus. This information is crucial for establishing an integrated action plan for continuous and ongoing education with a unified healthcare perspective.

Made all the changes as recommended.

Reviewer #2: (No Response)

Reviewer #3: Abstract:

Background: Rather than describing rabies as a significant global public health issue, characterize it in terms of endemicity, specifying the number of affected countries, considering that some are rabies-free. Use abbreviations consistently, such as Health Care Professional (HCP).
Done as directed (Rabies, caused by the rabdovirus, is a fatal zoonotic disease with over 59,000 annual deaths globally. Asia and Africa account for 95%, with India leading, followed by China. In Pakistan, where it's endemic, over 50,000 cases are reported yearly. Controlling rabid dog populations through vaccination is crucial in curbing mortality.)

As there is no definitive treatment, replace the terms "prevention and treatment" with "control and prevention" or "preventive treatment" in the wording. Revise the objective for clarity, avoiding the use of synonyms.

Done as directed.

In methods, address inconsistencies and eliminate redundancy. The method lacks representativeness, with unnecessary wording used to extend the text. For qualitative variables, use "association" instead of "correlation" in the context of describing the correlation.

Done as directed.

Maintain harmony: either use "healthcare professionals" or "healthcare workers." Please observe the use of uppercase letters.
Done as directed. (healthcare professionals).
In results, state that males were in preponderance, providing numbers and percentages. Justify the inclusion of faculty staff in the method section. Avoid starting sentences with numbers and use the full form for standard abbreviations and acronyms in subsequent text.

Done as directed.

Regarding "significant disparities in roles," the given text is ambiguous, and clarification is needed on what roles the author is addressing. Be consistent with the denotation of numbers (%) throughout the text.

Done as directed.

In conclusion, base recommendations on the study findings rather than on subjects that haven't been discussed.

Done as directed.

Introduction:
Please focus on describing the virus itself rather than its manifestations.

Done as directed.

Line 81: The statistics related to Asians and Africans cannot be supported by a single reference.

Removed.

The overall text predominantly centers around the incidence and prevalence. Although KAP results have been elucidated in relation to other nations, there is an abundance of published studies on the subject from this particular region. Furthermore, the transition to the implications of the study appears abrupt, lacking a thorough exploration of the rationale and existing gaps. It is imperative to provide a robust rationale for the study.
Methods:

The Methods section requires meticulous drafting. The term "design" should be clarified for precision.

Clarified.

Separate the questionnaire and consent, omitting redundancy.

Done.

Condense the study's duration by eliminating unnecessary wording and specify the exact timeframe.

Done.

Rationalize the selection of hospitals and clarify if the sample size was calculated.
Address the potential underpowered state of the study; with only 100 responses, confirm whether statistical power requirements were met or if the findings indicate a trend without statistical significance. If the study achieved the required power, explicitly mention it.
Specify the languages in which the questionnaire was administered and elaborate on the translation process for incorporating questions from other studies into the desired languages.
Clearly articulate the sampling technique and delineate the criteria for sample selection.
Include the ethical board approval number and reference for the questionnaires.
Specify whether the survey was conducted in English, Urdu, or both, and provide additional details on the questionnaire items and sections.

Done. (English)

Elaborate on the data analysis process, providing comprehensive details.

Done.

Methods:
Results: Please remove the first two lines (154, 155).
Please revise sentences 157-159, as they create the impression that the survey is conducted in a single hospital.

Done

I suggest deleting the sentence: "which may have ramifications for the findings of the research, presumably reflecting pre-existing gender inequalities in the healthcare sector." There is no need for possible explanations of out-of-context text.

Done.

Overall, the results are not up to the mark and require a complete revision.
The term "Training" given in Table 1 is ambiguous. Please clearly state its meaning. Moreover, the table is not formatted clearly; remove the percentage sign.
Utilize formal language instead of expressions like "to the best of our knowledge" to enhance the formality of the text.

Done.

Reviewer #1: The acronym (ARV) in table 2 is wrong:
Knowledge regarding availability of anti-rabies vaccine (AVR) in the vicinity of study hospitals.

Done as Directed.
In table 3, the values related to the variable “Rabies can spread through licks scratches” are wrong.
