# Supplementary material for: Knowledge, attitudes, and practices of healthcare professionals regarding rabies in tertiary care hospitals: A cross-sectional study in Peshawar, Pakistan
Source: PLoS Negl Trop Dis. 2024 Jun 10;18(6):e0012238. doi: 10.1371/journal.pntd.0012238 (PMC11192319; doi:10.1371/journal.pntd.0012238)
Supplement: Attachment — Submitted filename: Response to Reviewer.docx [file pntd.0012238.s003.docx]

Dear Editor in Chief,

I hope this message finds you well. I am writing to express my gratitude for the opportunity to submit a revised version of our manuscript titled "Knowledge, Attitudes, and Practices of Healthcare Professionals Regarding Rabies in Tertiary Care Hospitals: A Cross-Sectional Study in Peshawar, Pakistan" to PLOS Neglected Tropical Diseases.

We greatly appreciate the thorough review process conducted by the editorial board and the insightful feedback provided by the independent reviewers. Their comments have been invaluable in guiding our revisions and strengthening the quality of our manuscript.

In response to the reviewers' comments, we have made significant revisions to the manuscript. We have provided a detailed list of our responses to each comment and described the changes made in the revised manuscript. These revisions include clarifications on the roles of professional categories in patient care, explanations of the criteria for hospital selection, descriptions of the data analysis procedures, and improvements in the presentation of results and tables. Additionally, we have added a comprehensive discussion on the comparability among professional categories and the implications of knowledge gaps on treatment accessibility.

We believe that these revisions address the reviewers' concerns and significantly enhance the clarity and impact of our manuscript. We have submitted two versions of the revised manuscript: one with tracked changes and one clean version for ease of reading.

Thank you once again for considering our manuscript for publication in PLOS Neglected Tropical Diseases. We are hopeful that our revised manuscript meets the journal's standards and contributes meaningfully to the field of neglected tropical diseases.

We look forward to hearing from you regarding the outcome of the review process. Please do not hesitate to contact me if you require any further information or clarification.

Warm regards,

Bakhtiar

Corresponding Author

**Reviewer Comments**

**Response to Reviewer's Comment:**

Thank you for your perceptive remarks. We have included a segment that provides a concise overview of the responsibilities of the four professional groups engaged in our research, namely Medical Officers, Nurses, House Officers, and Paramedic Staff, specifically about the treatment of patients with rabies.

**Added Section to the Manuscript:**

**Roles of Professional Categories in Rabies Patient Care:**

Medical Officers have the main responsibility of doing the first evaluation and diagnosis of patients who have been injured by animals that have the potential to transmit rabies. The decision to provide rabies prophylaxis is made by evaluating the kind of exposure and the clinical symptoms. The nurses have a vital role in giving post-exposure prophylaxis, delivering wound care, and overseeing patients for any unfavorable responses to rabies immunoglobulin and immunizations, guaranteeing prompt treatment of such occurrences. House Officers provide support in the provision of emergency medical care and treatment to patients. They have the duty of promptly addressing wounds, evaluating the likelihood of rabies exposure, and commencing preventive therapy while being overseen by Medical Officers. Paramedics play a vital role in the pre-hospital environment by evaluating and treating patients at the location of an occurrence involving animal attacks. They guarantee the secure and expeditious transportation of patients to healthcare institutions for further treatment, while also conveying crucial information about the patient's state and the circumstances of their exposure to the medical team at the receiving end.

**Response to Reviewer's Comment:**

Thank you for your comment. We are grateful for the chance to elucidate the specific criteria used in the selection process of the three hospitals included in our research. We have included details explaining the reasoning behind the selection of these hospitals and their respective functions in the prevention and treatment of rabies.

**Added Section to the Manuscript:**

**Criteria for Selecting Study Hospitals:**

The selection of Lady Reading Hospital (LRH), Khyber Teaching Hospital (KTH), and Hayatabad Medical Complex (HMC) was determined by many features that make them particularly pertinent for research on the treatment of rabies exposure. The variables included are:

Reference Centers for Rabies Management: All three hospitals in the Peshawar area are important facilities for providing pre- and post-exposure rabies prophylaxis. The facilities are equipped with specialist resources to effectively manage and treat rabies, including the provision of rabies immunoglobulin and immunizations. This makes them crucial in the area healthcare system's reaction to instances of rabies.

High Patient Influx: These hospitals have a significant influx of persons seeking treatment for animal attacks, mostly because of their advantageous locations and the huge populations they cater to. The large number of patients coming in allows for a varied collection of healthcare interactions about the prevention and treatment of rabies.

Comprehensive Rabies Treatment Protocols: LRH, KTH, and HMC adhere to thorough rabies treatment practices that align with national and international recommendations. The strict adherence to defined protocols guarantees the uniformity and dependability of treatment and prevention measures, making these facilities ideal environments for examining practices and results.

The "Data analysis procedure" section is in the future tense. It wasn't described which statistical methods were used for data analysis. Were only frequencies and percentages used?

Done as directed


Results
Results are presented, and some of them are discussed in this section. I suggest using this section solely to describe the main findings of the research.

Done as directed.

The discussions in this section lead to suggestions that need to be reviewed considering only one variable. For example: Would it be possible to sustain an association between years of professional experience and lack of specific rabies training with potential deficiencies in readiness and understanding in describing the demographic characteristics of the participants?

Done as directed.

Tables 1 and 2 have categories that do not follow the same standardization. I suggest presenting the categories in descending order. For example: Professions of the Respondents.

Done as directed.

In the section "Association between respondents' work type and knowledge," I suggest replacing the word "association" with "relationship." No association measures were conducted.

Done as directed.

Regarding the results in Table 3, it would be interesting to focus according to the professional category of the respondents. Considering the education, years of study, and roles performed by each professional category in caring for people exposed to the rabies virus, would there be different response profiles?

Did the get what the reviewer is going to state?

In Table 3, it would be interesting to include the count (n) and percentage (%) for each response.

Done as directed.

I suggest including a discussion about the comparability among the professional categories included in the sample. Considering the level of education and years of study, are doctors and nurses comparable to paramedics and home officers?

Done as directed.

As a suggestion, for example, regarding the question "Does treatment prevent rabies in patients?" If nurses do not know, could it signify barriers to access adequate treatment? Similarly, if a paramedic does not know, would it be indicative of a barrier to access?

Answer accordingly.
